# Prediction of the birch pollen season characteristics in Cracow, Poland using an 18-year data series

**DOI:** 10.1007/s10453-012-9260-4

**Published:** 2012-05-11

**Authors:** Myszkowska Dorota

**Affiliations:** Department of Clinical and Environmental Allergology, Jagiellonian University Medical College, 31-531 Cracow, Sniadeckich 10 Poland

**Keywords:** Aerobiology, Birch, *Betula*, Predictive models, Meteorological parameters

## Abstract

The aim of the study was to construct the model forecasting the birch pollen season characteristics in Cracow on the basis of an 18-year data series. The study was performed using the volumetric method (Lanzoni/Burkard trap). The 98/95 % method was used to calculate the pollen season. The Spearman’s correlation test was applied to find the relationship between the meteorological parameters and pollen season characteristics. To construct the predictive model, the backward stepwise multiple regression analysis was used including the multi-collinearity of variables*.* The predictive models best fitted the pollen season start and end, especially models containing two independent variables. The peak concentration value was predicted with the higher prediction error. Also the accuracy of the models predicting the pollen season characteristics in 2009 was higher in comparison with 2010. Both, the multi-variable model and one-variable model for the beginning of the pollen season included air temperature during the last 10 days of February, while the multi-variable model also included humidity at the beginning of April. The models forecasting the end of the pollen season were based on temperature in March–April, while the peak day was predicted using the temperature during the last 10 days of March.

## Introduction

The genus *Betula* belongs to the Betulaceae family spread worldwide in the northern hemisphere, many times going beyond the moderate zone (Kornaś and Medwecka-Kornaś 2002). About 40 genera of birch occur in the northern hemisphere, mainly on the natural sites in mixed and pine forests (Seneta and Dolatowski 2007), whereas in the Mediterranean area, especially in the northern parts of Italy and Spain, birch trees are planted as ornamental trees (Méndez et al. 2005; Seneta and Dolatowski 2007).

The common occurrence of birches in Poland and allergenic features of pollen make birch the main source of tree pollen allergens provoking allergy symptoms in spring (from the middle of April to the beginning of May). Birch pollen allergens are responsible for allergic symptoms especially in Scandinavia and in Central Europe (D’Amato et al. 2007). In some regions of Europe, it was reported that the allergenic symptoms may be intensified both by pollen allergens from local sources and from the long-distance transport (Hjelmroos 1992; Skjøth et al. 2007; Siljamo et al. 2008; Veriankaite et al. 2010). The increase in rhinitis symptoms was also observed in some regions with the intensive birch cultivation (Troiste et al. 1996; Asero 2002). In Poland, about 10–15 % of people with pollen allergy are monosensitive to birch pollen allergens (Małolepszy et al. 2000; Samoliński et al. 2009). The research performed in Cracow showed that the allergy symptoms could also be caused by allergens occurring in the air out of the main pollen season (Madeja et al. 2005).

Most of the published results were obtained using the regression analysis preceded by determining the relationship between pollen season characteristics calculated by different percentage methods (Andersen 1991; Corden et al. 2002; Rodriguez-Rajo et al. 2003; Stach et al. 2008), cumulative methods (Drissen et al. 1990; Norris-Hill 1998; Groom-Adams et al. 2002; Laadi 2001b) or threshold values (Laadi 2001a; Radišic and Šikoparija 2005) and meteorological conditions (10-day mean values, mean monthly values, heat units, temperature accumulation). The beginning of pollen season, annual total and peak concentration are the most often predicted.

On the other hand, the analysis by Stach et al. (2008) determined the statistically significant influence of meteorological conditions in a year preceding the pollen season on the pollen season intensity, although the impact of NAO (North Atlantic Oscillation) circulation on the pollen season intensity was not indicated.

On the basis of the relationship between meteorological conditions and pollen season characteristics, the models predicting the birch pollen season concentrations were made in Poland only for Gdańsk (Latałowa et al. 2002).

The birch pollen season observations have been performed in Cracow since 1982. In 1982–1997, the gravimetric method was used, while since 1991, the volumetric method has been performed. The results of gravimetric method showed that the percentage birch of pollen in the annual total was the highest (Szczepanek 1994, 2006). Otherwise, the volumetric studies indicated the highest percentage of the birch pollen in the annual tree (Myszkowska et al. 2011), and also, the higher concentration of birch pollen in the city compared with the suburbs (Myszkowska et al. 2007).

Walanus (1994) analysing the gravimetric data in Cracow in 1983–1990 concluded that birch pollen rain was statistically correlated with most of the meteorological parameters except for wind velocity and cloudiness. The analysis of the pollen seasons of 15 taxa in Cracow pointed out that the highest daily concentrations were achieved in the first half of May, and they were caused by birch and pine pollen (Myszkowska et al. 2011). Analysing the birch pollen seasons in Cracow in 1991–2008, it was assumed that the relationship between the meteorological parameters (mean, minimum and maximum temperature) and the daily birch pollen concentration depends on the pollen season type (more or less dense). The influence of the local atmospheric circulation on the number of days with the daily concentration >80 PG/m^3^ (pollen grains per cubic metre) was also observed (Myszkowska and Piotrowicz 2009).

To analyse the problem of the birch pollen season forecast in Cracow, two aspects were considered: the common occurrence of birches in Poland and well-known allergenicity of birch pollen (Viander and Koivikko 1978; Moverare et al. 2002; Esch and Bush 2009).

The aim of the study was to construct the models forecasting the birch pollen season characteristics on the basis of meteorological conditions influencing the pollen season characteristics.

## Materials and methods

### Study site and climate

**Fig. 1:**
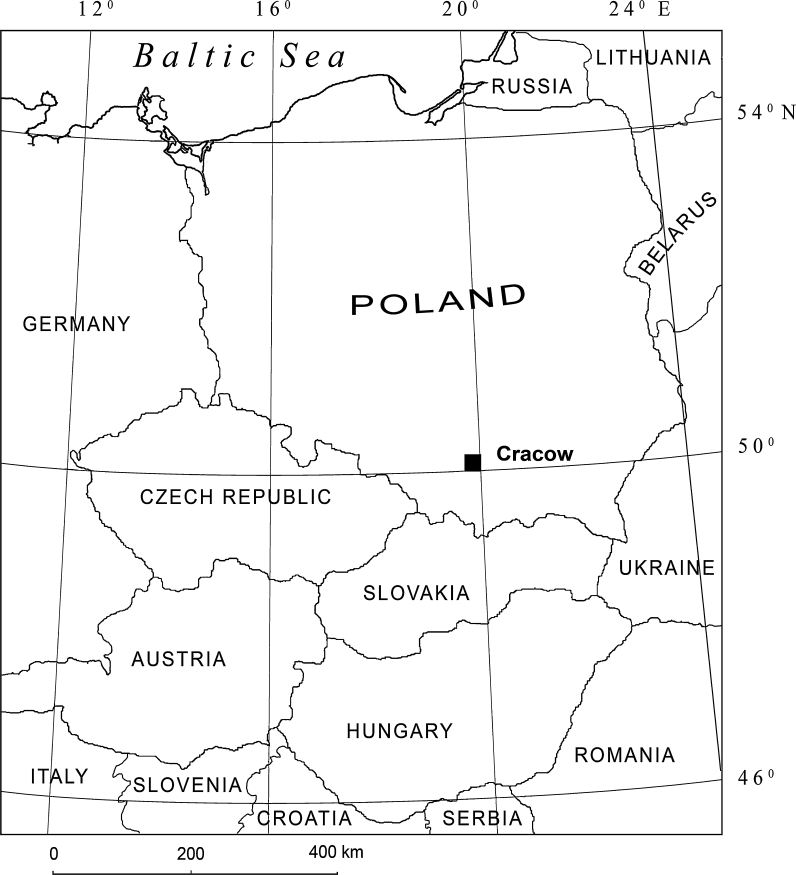
Study site location

Cracow (φ 50°04′N, λ 19°58′E, h 220 m a.s.l.) is located in the Małopolska province (Southern Poland) (Fig. 1), and considering the area and population, it is the second city in Poland. According to the data from 2010, the surface of the city is 327 km^2^; however, the population is 756,183 (1.98 % of the population of Poland). Cracow is the main university and cultural centre of Poland, housing 23 high schools. The private economic sector dominates (more than 80 % of income). The main fields of economic activity are as follows: industrial processing, building, trade and service industry (Raport o stanie Miasta 2010 2011) (http://www.bip.krakow.pl/dok_id=47152).

The city is surrounded by farmlands and forests. In Cracow, forests cover 4.23 % of the total city area. The broadleaf forests dominate making up 71 % of the total number of standing trees, with birch occurrence at 14 % (Turzański and Paula-Wilga 2002). To the north of Cracow, farmlands with small forest communities occur. To the south and east of Cracow, there are roughly equal areas of farmlands and forests. To the west of the city, forest communities prevail. The centre of Cracow is characterized by compact buildings surrounded by a green belt with *Aesculus* sp., *Tilia* sp*.*, *Acer* sp., *Populus* sp. and other ornamental trees and shrubs.

Generally speaking, in Poland, seven species of birch occur, the most frequent are as follows: *B. pendula* Roth (*B. verrucosa* Ehrh) and *B. pubescens* Ehrh. *B. pendula* is the most common species occurring in the whole country (Białobok 1979). In Cracow and its close surroundings, *B. pendula* dominates, while *B. pubescens* occurs occasionally in the Niepołomice Forest and towards the northwestern part of Cracow (Zając and Zając 2006).

Cracow is influenced by the air masses of the polar-maritime origin coming from over the Northern Atlantic, which bring thaw, an increase in cloud cover and snow in winter and cloud cover and rainfall in summer. The average air temperature in Cracow in the twentieth century was 8.7 °C, and 2000 was the hottest year (11 °C) (Piotrowicz 2007). The coldest month is January and the hottest July (with a monthly temperature of −2.1 and 18.9 °C, respectively). Sunshine duration per day is 3.9 h although from April to September it is 5.7 h (Woś 1999).

Annual precipitation is approximately 700 mm. The highest rainfall level is recorded in summer (June–August). In the annual cycle, about 40 % of rain falls in these months in Poland. Annual humidity is 79 %, and winds from a westerly direction prevail. Wind velocity is relatively low, about 2.9 ms^−1^ (Woś 1999).

In Cracow, a heat island occurs, of which intensity in the city centre reaches 1.2 °C on average. The heat island is responsible for a change in the thermal season duration during the year. For this reason, in the city centre, summer is longer by 25 days and winter is shorter by 23 days than in suburban areas. Higher temperatures in the city centre cause a longer vegetation season; the number of hot days (*t*_max_ > 25 °C) (Piotrowicz 2007) is higher by 10–11 days, and accumulated rainfall is higher than in the suburban area (Lewińska 2000).

### Meteorological data

Meteorological data were provided by the Research Station of the Dept. of Climatology, Institute of Geography and Spatial Management, Jagiellonian University (φ 50°04′N, λ 19°58′E, h 206 m a.s.l.). The weather observations have been carried out since 1792. The station is located in the immediate vicinity of the monitoring site. The parameters mean daily air temperature and mean relative humidity were calculated according to the instruction of the Institute of Meteorology and Water Management recommended for climatological stations in Poland (Lorenc and Suwalska-Bogucka 1995). To find the relationship between pollen season characteristics and meteorological parameters, the following meteorological parameters were used:

- Air temperature—as the initial data—minimum, maximum and mean daily temperature were taken. Mean daily temperature was calculated using the formula *t*_max_ + *t*_min_ + *t*_7_ + *t*_19_/4 (*t*_7_—temperature at 7 a.m., *t*_19_—temperature at 7 p.m.)
- Rainfall (mm)— “0” means lack of rain; “0.01” means trace of rain
- Relative humidity—mean value calculated using the formula (2 × *f*_7_ + *f*_13_ + *f*_19_)/4 (*f*_7_—humidity at 7 a.m., *f*_13_—humidity at 1 p.m., *f*_19_—humidity at 7 p.m.)
- Cloudiness (%)—mean value calculated on the basis of three measurements: at 6, 12 and 18 UTC. 100 %—full cloudiness, 0 %—cloudless sky. The scale 0–10 was used
- Relative sunshine—the number of sunshine hours in a given day related to the length of the day (hours from sunrise to sunset).

A 10-day mean and monthly mean of meteorological data were calculated. The characteristics of some meteorological parameters in the studied period (1991–2010) are presented in Table 1. The monthly average value is given in comparison with mean annual values.

**Table 1:** Descriptive characteristics of meteorological elements in Cracow in 1991–2010

Statistics	I	II	III	IV	V	VI	VII	VIII	IX	X	XI	XII	Annual mean
*Mean monthly temperature (* ^*o*^ *C)*													
$\bar{x}$	−1.2	0.1	3.7	9.3	14.6	17.9	19.8	18.9	13.6	8.9	3.8	−0.7	9.1
Min	−7.0	−4.7	−1.2	5.7	11.1	15.7	17.2	17.5	10.5	6.1	−0.9	−5.3	5.4
Max	4.1	4.3	6.6	12.2	17.2	19.7	22.4	21.9	16.2	12.5	7.2	3.2	1.3
SD	3.0	2.9	1.8	1.4	1.4	1.0	1.4	1.1	1.3	1.8	2.3	2.6	1.8
*Accumulative rainfall (mm)*													
$\bar{x}$	37.6	32.2	41.8	48.3	80.6	81.7	103.7	75.0	68.9	48.7	44.5	34.6	58.1
Min	13.3	5.5	16.2	0.5	31.6	10.6	32.3	20.9	27.6	6.8	16.5	13.8	37.3
Max	89.8	62.7	80.6	141.9	284.7	167.0	299.0	195.1	199.3	96.2	77.9	76.8	94.2
SD	18.2	14.4	20.1	32.9	55.2	40.5	65.6	43.2	43.8	29.5	18.1	14.8	12.6
*V*%	48.4	44.7	48.0	68.2	68.5	49.6	63.2	57.6	63.6	60.5	40.6	42.7	21.7
*Mean monthly relative humidity (%)*													
$\bar{x}$	82.6	79.9	74.6	67.4	68.2	69.2	70.6	73.3	79.8	82.9	85.1	84.9	76.5
Min	73.6	71.6	67.9	55.7	62.6	61.0	59.6	62.4	73.4	79.2	79.1	79.6	75
Max	86.8	84.7	80.3	74.3	82.9	80.5	81.6	81.1	88.6	86.5	89.2	88.8	79
SD	3.4	3.7	4.0	4.5	4.6	4.4	5.2	4.7	3.1	1.9	2.6	2.6	1.1
*V*%	4.1	4.7	5.3	6.6	6.7	6.4	7.4	6.5	3.8	2.3	3.0	3.0	1.5
*Mean monthly relative sunshine (%)*													
$\bar{x}$	18.6	22.6	28.1	39.2	44.3	43.6	47.6	47.8	37.2	30.2	18.5	15.2	32.7
Min	10.4	11.2	17.0	28.5	19.6	29.3	29.4	35.2	10.5	17.9	9.6	8.3	27.6
Max	25.2	35.2	39.4	67.9	59.9	56.7	74.3	62.5	53.8	43.9	30.3	28.5	37.0
SD	4.8	7.3	7.2	9.3	9.9	7.1	12.0	7.6	11.8	8.2	5.6	5.3	2.2
*V*%	25.7	32.2	25.6	23.7	22.4	16.4	25.3	15.9	31.7	27.2	30.0	35.0	6.8
*Mean monthly cloudiness (%)*													
$\bar{x}$	73.4	71.9	67.0	61.5	61.2	62.5	58.4	55.2	59.0	65.1	75.2	76.1	65.5
Min	59.3	59.2	53.0	33.8	46.0	47.0	29.7	37.1	34.7	50.3	62.5	66.4	59.2
Max	84.5	88.1	75.9	73.9	86.6	75.1	76.1	70.6	86.3	85.6	86.7	89.9	71.0
SD	7.7	8.0	7.8	9.4	10.2	8.2	12.2	9.2	13.3	9.0	6.5	6.7	2.9
*V*%	10.5	11.1	11.7	15.3	16.6	13.2	20.8	16.6	22.5	13.9	8.6	8.8	4.5

$\bar{x}$ arithmetic mean, *Min* minimum value, *Max* maximum value, *SD* standard deviation, *V*% coefficient of variation

### Birch pollen data

Birch pollen data were collected in Cracow using the volumetric method in 1991–2010. Two spore traps of the Hirst design (Hirst 1952) were used (Seven Day Recording Volumetric Spore Trap, Burkard Company in 1991–2003 and VPPS 2000, Lanzoni Ltd. in 2004–2010). The samplers were located on the roof of the Collegium Sniadeckiego building in the city centre 20 m above ground level. The samples collected by both samplers are comparable, because of the same technical conditions and parameters, mainly of air flow of 10 l/min. Pollen grains were sucked in on a rotating drum covered by transparent tape (*Melinex tape*) with an adhesive fluid. The preparation of microscopic slides was made according to the instruction by Stach and Kasprzyk (2005). The tape with an adhesive fluid was changed once a week and then divided into seven segments corresponding to 24-h periods. The samples were examined using a light microscope at 400× magnification. Pollen grains were counted along 4 longitudinal transects in 2000–2010 (method recommended by the Spanish aerobiological network (REA) (Galán et al. 2007), and earlier in 1991–1999, the 12 traverse transects method was employed. According to Cariñanos et al. (2000), the proportion of analysed surfaces is 11.25 % of the total surface for the transverse method and 12.85 % for the longitudinal method. Birch pollen counts are expressed as pollen grains per cubic metre (PG/m^3^). The four longitudinal method is also used according to the European Aerobiology Society rules of quality control (Šikoparija et al. 2011).

On the basis of the daily birch pollen concentrations, the diagram of daily distribution was prepared. The basic statistics, like arithmetic mean, standard deviation, minimum, maximum, were considered. Birch pollen seasons were calculated by the percentage method. Having compared the different percentage methods, like 90 % (Nilsson and Persson 1981; Latałowa et al. 2002), 95 % (Andersen 1991; Rodriguez-Rajo et al. 2003; Stach et al. 2008) and 98 % (Emberlin et al. 1993), the combined 98/95 % method was chosen. The number of days with “no” pollen grains before and after the dense pollen occurrence, the value of pollen concentration in the first days with pollen grains, the value of coefficient of variation and also other author suggestions (Jato et al. 2006) were taken into consideration. Using this method, the beginning of pollen season was calculated as 1 % of annual total and the end of pollen season as 97.5 % of annual total, what resulted in exclusion of the days with low pollen concentration. The SPI value means seasonal pollen index is determined as a seasonal total concentration in the determined season. While the maximum concentration is the highest, daily pollen concentration observed during the season.

### Statistical analysis

Descriptive statistics were used to estimate pollen season characteristics and also to compare different methods of pollen season calculations. The Spearman rank correlation test was used to find the relationship between the pollen season characteristics and meteorological parameters (STATISTICA program version 9.0). Only the statistically significant correlation coefficients were presented (the correlation coefficients were significant at the 0.01 level).

The predictive models were calculated on the basis of backward stepwise multiple regression analysis for the beginning of pollen season, the end, the peak day, peak concentration and SPI value (the annual pollen total calculated in the pollen season). To prepare the models for SPI value prediction, the meteorological data from the given year and the year preceding the year of observations were used, especially from June to August.

Both multi-variable models and single-variable models were tested using the data in 2009 and 2010. The presented models contain the explanatory variables, which are not correlated with each other and explained the majority of variation of dependent variable. Both models with and without an intercept were tested. The prediction errors were determined as a difference between observed and expected values on the basis of a given model. SAS program version 9.2 was used to generate the predictive models and estimate the accuracy of the models.

## Results

### Analysis of pollen seasons

**Fig. 2:**
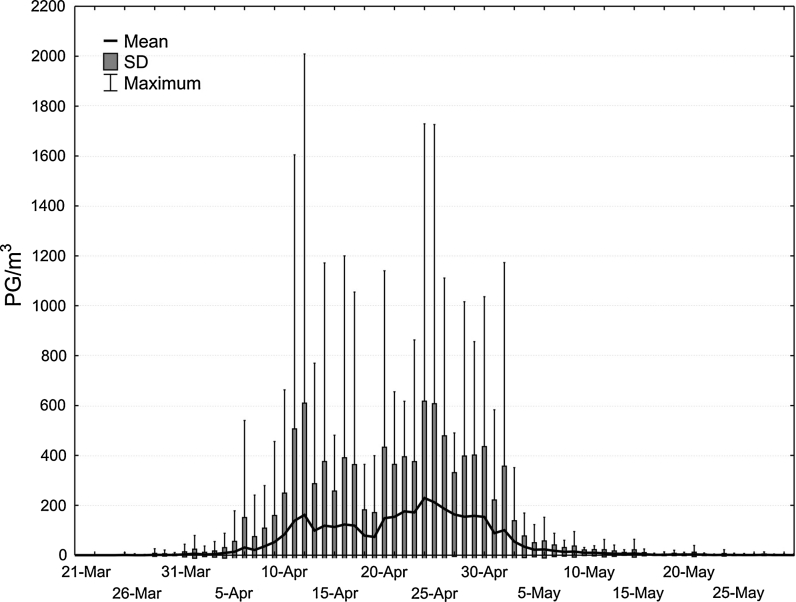
Distribution of daily birch pollen concentrations (15 March–30 May). On the basis of 20 data (1991–2010), arithmetic mean concentration and standard deviation are calculated

In Cracow, in the period studied, the first birch pollen grains appeared at the beginning of April, but they could be observed from the 6th of March to the 23rd of April (Fig. 2). Birch pollen grains occur in a dense way from the beginning of April to the middle of May, although the single pollen grains are observed even in October. They are rarely noted before the dense pollen season.

**Fig. 3:**
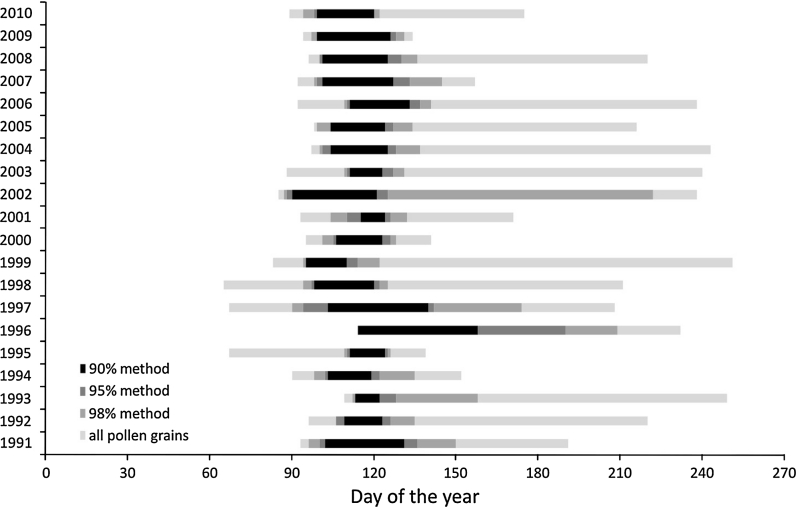
The dates of the start and end of birch pollen seasons in Cracow in 1991–2010 presented as all pollen grains occurrence and calculated by the following methods: 98, 95, 90 %

The pollen season calculated by the 98/95 % method started approximately on the 10th of April (101st day of the year), the earliest pollen season started in 2002 (the 28th of March), the latest in 1996 (the 23rd of April). The mean pollen season duration was 31 days (ranging from 17 to 77 days). Comparing the time of all pollen grains occurring and the percentage methods, it was indicated that the season time is shortened by more than 50 % (Fig. 3).

**Fig. 4:**
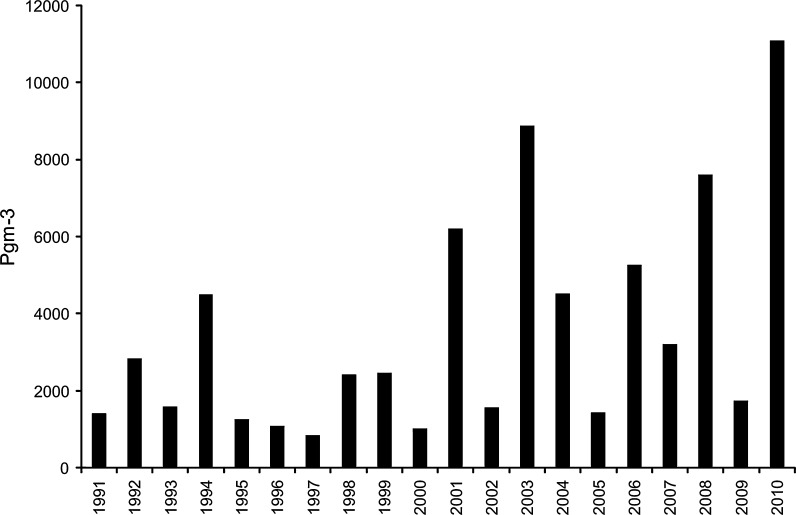
Birch seasonal pollen index (SPI) in Cracow in 1991–2010 calculated by 98/95 % method

Peak concentration was weakly correlated with the SPI value (*r* = 0.559; *p* < 0.05), and the highest SPI value was gained in 2010 (11 099 PG/m^3^). No increasing or decreasing trend of SPI value was noted, and the years of higher intensity were interrupted by the lower ones (Fig. 4).

The most variable pollen season parameter calculated using the 98/95 % method was the SPI value (*V*% = 82.18) (Table 2). Also, the peak concentration was really variable (*V*% = 80.62), in comparison with the peak day (*V*% = 6.24) (Table 2), which differed from the mean value only about 6 days (the 18th of April). The beginning of pollen season was less variable in comparison with the end (*V*% = 7.36 and *V*% = 11.71, respectively).

**Table 2:** Coefficient of variation (*V*%) calculated for different methods of birch pollen seasons calculation in Cracow in 1991–2010

Method/parameter	Season start*	Season end*	Season duration**	SPI***
98 % Method	7.36	18.91	63.83	81.85
95% Method	6.83	11.82	49.74	75.72
90 % Method	6.44	7.63	40.58	82.39
98/95 % Method	7.36	11.71	44.84	82.18
Peak day*	6.24	Peak concentration***	80.62	

98 % Method—season start calculated as the first day when the concentration reaches 1 % of annual total; season end calculated as the last day with the concentration reaching 99 % of annual total; 95 % method—season start calculated as the first day when the concentration reaches 2.5 % of annual total; season end calculated as the last day with the concentration reaching 97.5 % of annual total; 90 % method—season start calculated as the first day when the concentration reaches 5 % of annual total; season end calculated as the last day with the concentration reaching 95 % of annual total

* Day of the year from the 1st January; ** number of days; *** pgm^−3^

The last two pollen seasons (2009 and 2010) were classed as less-dense pollen seasons according to the results reported by Myszkowska and Piotrowicz (2009). Both pollen seasons started in the first week of April, lasted more than 20 days and the peak concentrations differed strongly (173 PG/m^3^ in 2009; 1,200 PG/m^3^ in 2010).

### The relationship between pollen season characteristics and meteorological parameters

The results of the relationship between birch pollen season characteristics and meteorological parameters were presented for the long data series 1991–2008 (Tables 3, 4). The start of pollen season was strongly related to mean temperature before the calculated pollen seasons, especially by a 10-day mean temperature in the last week of February and the first week of March the strongest. The start of pollen season was also related to the rainfall in the last week of March and cloud cover in March.

**Table 3:** Significant Spearman’s correlations between birch season start end and meteorological conditions in Cracow in 1991–2008

	Correlation coefficient
*Dependent variable: season start*	
10-Day mean daily average temperature in 51–60 days from 1 January	−0.702
10-Day mean daily average temperature in 61–70 days from 1 January	−0.702
Mean March daily average temperature	−0.579
Mean February–March daily average temperature	−0.487
Mean January–March daily average temperature	−0.492
10-Day mean daily rainfall in 81–90 days from 1 January	0.572
10-Day mean daily average relative humidity in 91–100 days from 1 January	0.510
Mean March daily relative humidity	0.563
Mean March daily cloudiness	0.561
*Dependent variable: season end*	
Mean January–February daily average temperature	−0.538
Mean March–April daily average temperature	−0.680
Mean February–April daily average temperature	−0.491
Mean January–March daily average temperature	−0.535
Mean January–April daily average temperature	−0.540
10-Day mean daily rainfall in 21–30 days from 1 January	−0.479
10-Day mean daily rainfall in 141–150 days from 1 January	0.487
Mean February–April daily average rainfall	−0.511
Mean January–April daily average rainfall	−0.484
10-Day mean daily average relative sunshine in 161–170 days from 1 January	0.581
10-Day mean daily average relative humidity in 161–170 days from 1 January	−0.581
10-Day mean daily average cloudiness in 121–130 days from 1 January	0.593
10-Day mean daily average cloudiness in 161–170 days from 1 January	0.581

Correlation is significant at the 0.01 level (2-tailed)

**Table 4:** Significant Spearman’s correlations between peak concentration, peak day and SPI value of birch pollen seasons and meteorological conditions in Cracow in 1991–2008

	Correlation coefficient
*Dependent variable: peak concentration*	
Mean February daily average rainfall	−0.527
10-Day mean daily average relative sunshine in 31–40 days from 1 January	−0.542
Mean January–February daily relative humidity	0.513
10-Day mean daily average cloudiness in 41–50 days from 1 January	0.509
*Dependent variable: peak day*	
10-Day mean daily average temperature in 1–10 days from 1 January	−0.535
10-Day mean daily average temperature in 21–30 days from 1 January	−0.524
10-Day mean daily average temperature in 51–60 days from 1 January	−0.515
10-Day mean daily average temperature in 81–90 days from 1 January	−0.510
10-Day mean daily average temperature in 91–100 days from 1 January	−0.576
10-Day mean daily average temperature in 101–110 days from 1 January	−0.616
Mean January daily average temperature	−0.606
Mean January–February daily average temperature	−0.502
Mean January–March daily average temperature	−0.548
10-Day mean daily rainfall in 21–30 days from 1 January	−0.528
10-Day mean daily rainfall in 81–90 days from 1 January	0.506
10-Day mean daily average cloudiness in 61–70 days from 1 January	−0.516
Mean February daily average cloudiness	−0.475
*Dependent variable: SPI*	
10-Day mean daily relative sunshine in 31–40 days from 1 January	−0.513
Mean February daily relative humidity	0.501
Mean January–February daily relative humidity	0.486
10-Day mean daily average cloudiness in 31–40 days from 1 January	0.507

Correlation is significant at the at the 0.01 level (2-tailed)

The end of pollen season was influenced by more meteorological parameters compared with the beginning. The statistically significant relationship between the pollen season end and mean monthly temperature from January to April was found. Rainfall in the first decade of the year and at the end of April and relative sunshine in the middle of May delayed the end of pollen season. Also the relative humidity in the third week of June and cloudiness in the second week of April and in June delayed the end of pollen season.

Peak concentration was influenced by rainfall in February (negative correlation), relative sunshine at the beginning of February and relative humidity in January–February, while the influence of temperature on peak concentration was not found. More meteorological parameters influenced the day of peak concentration. The temperature in the whole period before pollen season influenced the peak day the strongest (negative correlation), as well as the rainfall in the last week of January, relative sunshine and cloudiness in February.

It was stated that the meteorological parameters in the year of observations influenced the SPI value, although in the year preceding the year of observations, the influence of humidity in November and December was reported (Myszkowska 2010). In the 1991–2008 series, the influence of relative sunshine and cloudiness at the beginning of February was found. The obtained correlation coefficients between the SPI value and the meteorological parameters from the year preceding the year of observation were not statistically significant.

### Predictive models

The backward stepwise multiple regression analysis was applied to generate the regression models that fitted the observed data the best. The models concerning one to three dependent variables were finally chosen. For the models presented in Table 5, the regression coefficients and prediction errors were calculated. Because of the very low determination coefficients calculated for SPI models and the highest prediction errors, the models were excluded from the analysis. The estimation of the models predicted the peak concentration value was also not fully satisfied, although the models containing two independent variables are better fitted. The accuracy of the models predicting the start of pollen season was higher in 2009 compared with 2010. The predictive models constructed by using the pollen season characteristics and the meteorological parameters fitted the pollen season start and end the best, especially models containing two independent variables in 1991–2008.

**Table 5:** Models created for the following season characteristics: season start, season end, peak concentration and peak day, in Cracow, in 1991–2008

Season parameters vs meteorological elements and models	*R* ^2^	Observed 2009	Expected 2009	Difference	Observed 2010	Expected 2010	Difference
*Season start*							
Temp_6_ *t* = −3.551, *p* = 0.003; Hum_10_ *t* = 2.864, *p* = 0.012 SS = 61.55 − 1.34*Temp_6_ + 0.59*Hum_10_	0.620	97	98	−1	94	99	−5
Temp_6_ *t* = −3.352; *p* = 0.004 SS = 102.94 − 1.51*Temp_6_	0.412		104	−7		97	−3
*Season end*							
Temp_M23_ *t* = −3.864, *p* = 0.003; Rain_15_ *t* = 2.832, *p* = 0.013 SE = 168.86 − 7.27*Temp_M23_ +3.35*Rain_12_	0.649	128	129	−1	120	126	−6
Temp_M23_ *t* = −3.705, *p* = 0.002; SE = 184.12 − 8.23*Temp_M23_	0.462		121	7		128	−8
*Peak concentration*							
Cloud_6_ *t* = −3.015, *p* = 0.009; *t* = 2.453, *p* = 0.027 PC = −1,337.96 26.45*Cloud_6_ + 48.02*Hum_4_	0.485	173	831	−658	1,200	641	559
Cloud_6_ *t* = −2.488, *p* = 0.024 PC = 2,414.9 − 24.95*Cloud_6_	0.279		502	−329		866	334
*Peak day*							
Temp_9_ *t* = −3.507, *p* = 0.003; Temp_6_ *t* = 2.504, *p* = 0.024 PD = 121.85−2.16*Temp_9_−0.97*Temp_6_	0.524	107	113	−6	106	97	9
Temp_9_ *t* = −2.776, *p* = 0.014 PD = 119.65 − 1.96* Temp_9_	0.325		111	−4		100	6

The characteristics of the statistically significant relationship between meteorological parameters used in the models and the season characteristics

Temp_6_—mean daily temperature in 51–60 days from 1.01; Temp_9_—mean daily temperature in 91–100 days from 1.01; Temp_M23_—mean March–April temperature; Temp_M4_—mean April temperature; Rain_12_—mean rainfall in 111–121 days from 1.01; Hum_4_—mean daily average relative humidity in 31–40 days from 1.01; Hum_10_—mean daily average relative humidity in 91–100 days from 1.01; Cloud_6_—mean daily cloudiness in 51–60 days from 1.01

*SS* season start, *SE* season end, *PC* peak concentration (PG/m^3^), *PD* peak day

The multi-variable models predicting the start of pollen season contained temperatures in the last week of March and humidity in the first week of April. In spite of the fact that multi-variable models fit the pollen season start weaker, they seem to be more effective. On the other hand, the one-variable model including temperature in the last week of February fitted weaker.

## Discussion

Most of the forecasting models used in aerobiology are based on the defined relationship between pollen season characteristics and meteorological parameters (Norris-Hill 1998; Laadi 2001b; Stach et al. 2008). The percentage method seems to be less appropriate for the pollen season start calculation compared with the cumulative method (Norris-Hill 1998), because of a strong relationship between the start of pollen season and pollen season severity. On the other hand, in case of other pollen season characteristics prediction, the percentage method is more useful. Moreover, the percentage method cuts the days with “0” pollen grains or with very low concentration, which let us not to extend the pollen season duration. The disadvantage of these methods is that the pollen season could be calculated only when the pollen occurrence is over (Stach and Kasprzyk 2005; Jato et al. 2006; Piotrowska 2007).

The different percentage methods are used to calculate the birch pollen season (Andersen 1991; Emberlin et al. 1993; Nilsson and Persson 1981; Latałowa et al. 2002; Kasprzyk 2003; Stach et al. 2008). In this paper, the 98/95 % method was proposed, allowing us to cancel days with zero concentrations in the post-peak period and to include the days with the birch threshold concentration into analysis (Stach et al. 2008).

The birch pollen season characteristics variation pointed out that the start of pollen season was the least variable parameter, followed by the end and peak day. In relation to the meteorological parameters, the birch pollen season start fluctuates about two weeks from the mean value. However, the strong variation in SPI value and peak concentration depending both on the meteorological parameters and birch biological cycle was reported by Laadi (2001a) and Latałowa et al. (2002).

Comparing the results obtained by other authors, for example Laadi (2001a), the start of birch pollen season depends mainly on temperatures before the pollen season, which was confirmed also in Cracow, where the temperature in the third week of February and the first week of March (about four weeks before the mean pollen season start) influenced the pollen season start the strongest. Sarvas (1967) reported that the birch pollination in Poland, Germany, Czech Republik, Slovakia and Finland starts after the temperature achieved the 4.1–5.4 % of average annual temperature over 5 °C. It is assumed that the thermal conditions up to 30 days before the pollination influence the time of pollen grains occurrence the strongest.

Including only temperature into the models preparation seems to be insufficient, which was stressed by some authors (Méndez et al. 2005). In the present paper, the models including temperature in 51–60 days from the 1st January and temperature in February–March fitted the start of pollen season the best. Having included the additional independent variables into the model, like relative humidity in 91–100 days from the 1st January and rainfall in March, a decrease in *r* ^2^ value was observed. The expected parameters indicated the later pollen season start date than observed values showed. Some authors reported that the relative sunshine or photoperiod have a significant impact on the start of birch pollen season (Andersen 1991), but in our study, it was not confirmed.

The end of birch pollen season in Cracow seemed to be more stable than the beginning, although Jäger et al. (1996) reported that in Northern Europe and in Austria, the significant trend towards earlier pollen season ends was observed in 1980–1995. Also Spieksma et al. (1995) showed some variation in pollen season starts in different parts of Europe.

The rainfall in the last week of April (just before the mean pollen season ends) influenced this characteristic of pollen season in Cracow the strongest. In spite of that the models prepared on the basis of 1991–2008 series seemed to be more effective when temperature in March and April was included, the highest temperature was in that time the faster the pollen season end was observed. It is assumed that intensive birch pollen release from the anthers takes place when thermal conditions are favourable. The stronger accuracy was observed for the models containing two independent variables (temperature in March and April and rainfall in the last decade of April). It could be explained by pollen behaviour in increasing temperature and rainfall, which extends pollen release in all birch trees occurring in the studied area.

The peak day does not depend on the pollen season calculation. It seems that this parameter should be closely related to the start of pollen season, but in the case of the present study, this phenomenon was not confirmed. On the other hand, the birch pollen is released intensively during two weeks, and up to 80 % of pollen is released by the first 2–3 days from the start of pollination (Białobok 1979). The examples of the confirmed relationship between the start of birch pollen season and peak day have not been found, while this relation in case of grass pollen seasons was reported by Garcia-Mozo et al. (2009). If the peak day was strongly related to the beginning of pollen season, it would be possible to predict the peak day easily on the basis of the pollen season start day. Unfortunately, because of several birch species influencing the daily pollen concentration, the peak day is often observed as the highest concentration among very high concentrations. There are only a few papers referring to the peak day forecasting, for example Laadi (2001a), who used a multiple component analysis to predict a day of high allergenic risk.

In spite of the close relationship between the peak concentration and SPI value, the different meteorological parameters determined these pollen season characteristics. Both single- and two-variable models weakly fitted the maximum concentration. The created models can predict no more than 50 % of the observed value. What was really interesting, in most days in 2009 the expected values were lower than the observed ones; however, in 2010, the situation was reversed. Presumably, this observation could be associated with the considerably higher SPI value and peak concentration in 2010. The previous results obtained in Cracow indicated the relationship between the SPI value and the meteorological parameters in the year before observations (Stach et al. 2008; Myszkowska 2010). The current study did not indicate a statistically significant relationship between the SPI value and the meteorological parameters in the preceding the year of observation. For this reason, the models for the SPI value prediction were not considered in this paper.

As other authors suggested (Jato et al. 2007; Linkosalo et al. 2010), the phenological models could be more efficient for the prediction of pollen season intensity. The estimation of the models for peak concentration, including the cloud cover in the middle of February, was comparable to the models for SPI value. Temperature did not determine this characteristic. Also, Dahl and Strandhede (1996) suggested, analysing *Betula alba* flowering pollen seasons, the intensity of the pollen season is dependent on the amounts of assimilates, on the potential number of sites, which are important for catkin initiation during the previous year, and it is also dependent on the weather during pollen dispersal.

In conclusion, the prediction of the birch pollen season characteristics on the basis of regression analysis seems to be an effective tool for some of the birch pollen seasonal parameters forecasting. The constructed models fitted the start of pollen season and peak day the best. Temperature before the pollen season was recognized as the main meteorological parameter, determining most of the pollen season characteristics, except at peak concentration. The models forecasting the peak concentration are of low importance as pollen season predictors. Also, the prediction of the SPI value on the basis of meteorological parameters only did not give the expected results.
